# Open, High-Resolution EI+ Spectral Library of Anthropogenic Compounds

**DOI:** 10.3389/fpubh.2021.622558

**Published:** 2021-03-09

**Authors:** Elliott J. Price, Jirí Palát, Katerina Coufaliková, Petr Kukučka, Garry Codling, Chiara Maria Vitale, Štěpán Koudelka, Jana Klánová

**Affiliations:** ^1^Faculty of Sports Studies, Masaryk University, Brno, Czechia; ^2^RECETOX Centre, Masaryk University, Brno, Czechia

**Keywords:** electron ionization [EI+], spectral library, gas chromatography mass spectrometry, chemical exposure, high-resolution

## Abstract

To address the lack of high-resolution electron ionisation mass spectral libraries (HR-[EI+]-MS) for environmental chemicals, a retention-indexed HR-[EI+]-MS library has been constructed following analysis of authentic compounds via GC-Orbitrap MS. The library is freely provided alongside a compound database of predicted physicochemical properties. Currently, the library contains over 350 compounds from 56 compound classes and includes a range of legacy and emerging contaminants. The RECETOX Exposome HR-[EI+]-MS library expands the number of freely available resources for use in full-scan chemical exposure studies and is available at: https://doi.org/10.5281/zenodo.4471217.

## Introduction

Since commercial release in 2015, the high-resolution gas chromatography Orbitrap mass spectrometer (GC Orbitrap MS) has been evidenced as a valuable tool in metabolomics (1–6), environmental (7–10), clinical (11), and forensic analysis (12). The enhanced mass accuracy and greater achievable linear dynamic range (13), when operating in full-scan mode, makes GC Orbitrap MS particularly suited to chemical characterization of complex samples with unknown composition.

The most commonly applied ionization for screening of environmental contaminants is electron ionization (EI+), typically operated at 70 electron volts (eV), favored for robust fragmentation. When coupled with retention index information, the matching of EI+ spectra can enable structural annotation of relatively high confidence (14).

However, high-resolution electron ionization mass spectral (HR-[EI+]-MS) libraries are currently limited (1), particularly for environmental chemicals. This hinders the application of GC Orbitrap MS without prior generation of in-house spectral libraries, which requires substantial resources; or the purchase of commercial libraries that are often tied to proprietary data formats and software.

Whilst matching to low resolution (LR) spectra is possible and additional accurate mass information utilized [e.g., via High Resolution Filtering (15) (HRF)], freely available LR-[EI+]-MS libraries are equally limited in coverage of environmental chemicals. Furthermore, scan time has a significant impact of fidelity of isotopic abundance (16) and specific chemical gas-phase reactions in the trap (17) can lead to Orbitrap system-specific spectra. In addition, it is known that spectra are source-dependent, even at standardized 70 eV (18). These additional information are needed to be overlooked when matching to LR-[EI+]-MS spectra and entail discrepancy to current spectral predictions and substructure characterizations.

Accurate spectral prediction is particularly crucial for the generation of “suspect” libraries for GC-[EI+]-MS screening of environmental contaminants, to improve identification of unknowns (19). The lack of [EI+]-MS spectra for environmental chemicals is a constraint for spectral prediction (20) by limiting inputs for machine-learning methods and preventing validation of computed spectra.

Herein, the RECETOX Exposome HR-[EI+]-MS library has been generated for free distribution to enable accelerated application of GC-Orbitrap MS for identification of environmental contaminants.

## Materials and Methods

### Chemicals

All reagents were of GC grade (for pesticide residue analysis) or higher. Standards were of ≥98% purity and stored as per manufacturer recommendations. Compounds were selected on the basis of being included in targeted environmental and biomonitoring analysis undertaken or under method development by the RECETOX Trace Analytical Laboratories (under EN ISO/IEC 17025:2005 accreditation), thus with known amenability for GC-[EI+]-MS. Standards were purchases in solution form and dilutions conducted following accredited trace analytical laboratory practice. Where necessary, solvent was switched to pyridine or hexane, under high purity N_2_. Individual aliquots of 50 μL were transferred into 2 mL amber vials with built-in 350 μL insert and stored at −20°C prior to injection.

### Data Acquisition

Compounds or compound mixes were analyzed via GC Orbitrap MS comprising a Trace 1310 Series GC, Q Exactive GC-Orbitrap MS and TriPlus RSH Autosampler. Injections (1–2 μL, providing >100 pg on column per analyte) were made in splitless mode using split/splitless injector. Separation was performed on a 5-type MS column (30 m × 0.25 mm, 0.25 μm i.d.; cross-linked 5% phenyl-95% methylpolysiloxane, Restek Rxi-5Sil MS) with guard (1 m × 0.53 um i.d.; non-polar deactivated fused silica, Restek Rxi guard) with helium as carrier gas (1.3 mL/min). The Orbitrap MS was operated in Full MS-SIM using 70 eV EI+ and data recorded in profile mode, scan range 70–700 m/z. Filament emission was 50 μA, MS transfer line at 250°C, and ion source at 280°C. Resolving power was 60,000 full-width at half maximum height at m/z 200, automatic gain control at 1E6 and automatic max injection time. A C_7_-C_40_ alkane series was used for external non-isothermal Kováts retention-indexing (from temperature programming, using the definition of Van den Dool and Kratz) (21).

### Library Construction

Vendor raw files were converted to mzML format using ProteoWizard MSConvert (22, 23) (ver 3) with vendor centroiding. Component peak identification and spectral deconvolution was performed using MS-DIAL (24, 25) (ver 4.20). Parameters were set as follows: minimum peak height: 50,000; mass slice width: 0.05; mass centroiding accuracy: 0.05; average peak width: 10; smoothing level: 3; sigma window: 0.3 and 1% spectra cut-off. Quality of deconvoluted spectra was manually checked (26) and acceptable spectra exported to MS-FINDER (25, 27) (ver 3.42). Precursor m/z was assigned as nearest ion in the spectra equal to or less than the compounds monoisotopic mass and fragments were annotated with a (5 ppm) tolerance. Spectra were saved in the MS Transfer File (MSP) format with a 1% relative abundance cut-off (28). Retention indices (RI) were retrieved from MS-DIAL and input to the MSP. Where possible, spectra were verified via similarity matching (forward search) (29) against LR-[EI+]-MS spectra of a composite library comprising NIST/EPA/NIH MS Library (NIST 14) (30), MS-DIAL MSP spectra kit of public EI-MS spectra (25, 31) (ver 2), SWGDRUG MS library (32, 33) (ver 3.6), Cayman Spectral Library (34) (v09112019), and Golm Metabolome Database (35) (v20112021). RIs were compared to consensus semi non-polar RIs (36). Spectral and RI matches were conducted via NIST MS Search (ver 2.3) (37), constrained to the 70–700 m/z scan range.

### Database Management

Compound identifiers (InChI, InChIKey & SMILES) were retrieved via the Chemical Translation Service (38) (chemical name as input), United States Environmental Protection Agency (EPA) CompTox Chemicals Dashboard (39) (ver 3.5, chemical name and/or InChI as input) or generated in ACD/Chemsketch (40) (manually drawn structure). Predicted physico- and toxico-chemical properties were retrieved from the EPA CompTox Chemicals Dashboard (39) (ver 3.5, InChIKey as input); or generated via DataWarrior (41) (ver 5.2.1, SMILES as input). Natural product likeness scores were calculated via NP-Scout (42) on the NERDD portal (43) (SMILES as input). Structural classification was calculated via ClassyFire (44) (SMILES as input). Distribution plots were generated using plotly online (45) (available at https://chart-studio.plotly.com/). The database was compiled and exported in structure data formation (SDF) through DataWarrior (41).

## Results And Conclusions

Authentic compounds have been analyzed in full-scan mode using GC-Orbitrap MS and the constructed RECETOX Exposome HR-[EI+]-MS library incorporates GC retention-index (alkane series, semi non-polar column) and theoretical fragment formula annotation.

The library contains compounds of broad physicochemical diversity (Table 1, Supplementary Figure 1) and toxicological importance. Of the 386 spectra collected, 336 are unique to the RECETOX Exposome HR-[EI+]-MS library with respect to the 31,491 contained in other freely available libraries (composite library excluding NIST 14; Supplementary Table 1). Notably, the majority of compounds (318 of 352) are listed on the Human Biomonitoring for Europe (HBM4EU) Screening List for Chemical of Emerging Concern (CECscreen) (47) (Supplementary Table 1).

**Table 1:** Overview of current diversity of compounds included within HR-[EI+]-MS library.

Number of spectra	386
Unique compounds^a^ (connectivity)	351
Number of chemical classes^b^	56
Monoisotopic mass range	108.06–715.45
boiling point (^°^C) range^c^	170.51–549.62
Octanol water coefficient (log*P*) range^c^	−0.84–9.20

a *Compounds with unique connectivity (46)*.

b *ClassyFire taxonomy (44)*.

c *Predicted values retrieved from the EPA CompTox Chemicals Dashboard (39)*.

The MSP format is widely used, readable and modifiable by commercial and freely available software tools (48), enabling easy incorporation into current annotation workflows. Spectral quality was ensured and comparison of the HR-[EI+]-MS entries to LR-[EI+]-MS libraries generated an average forward match score of 841 (Supplementary Table 1). In use, adequate spectral matches to the HR-[EI+]-MS library enhanced with RI match on similar 5-type semi-non polar columns (49) would warrant a level 2 “putative” annotation (50, 51) (exampled in Supplementary Figure 2). Furthermore, the high degree of compound diversity is beneficial for integration with EI+ spectral similarity networking via GNPS-MSHub (52) online workflows or offline via MetGEM (53) to assign compound class (level 3 annotation) (54).

The accompanying SDF database accompanies structures with structural classifiers and predicted physico- and toxico- chemical properties. Sharing facilitates ease of insight into chemical properties (exampled in Supplementary Figure 3) and future use of compound data for modeling, e.g., retention prediction (55).

The RECETOX Exposome HR-[EI+]-MS library is freely provided to enable broad usability and promotes open science in environmental research (56). We hope the RECETOX Exposome HR-[EI+]-MS library provides a valuable resource for those seeking to screen environmental exposures and chemical contaminants.

## Data Availability Statement

The RECETOX Exposome HR-[EI+]-MS library and accompanying compound database are available for download at: https://doi.org/10.5281/zenodo.4471217.

## Author Contributions

EJP devised the concept, generated the individual spectral files, compiled the spectral library and compound database, drafted the original, undertook editing, and review of the manuscript. JP and KC generated the individual spectral files, curated the spectral library, and reviewed the manuscript. PK and GC oversaw the chemical management, generated the individual spectral files, and reviewed the manuscript. CV and ŠK curated the compound database and reviewed the manuscript. JK secured the funding, edited, and reviewed the manuscript. All authors contributed to the article and approved the submitted version.

## Conflict of Interest

The authors declare that the research was conducted in the absence of any commercial or financial relationships that could be construed as a potential conflict of interest.
