# Supplementary material for: Open, High-Resolution EI+ Spectral Library of Anthropogenic Compounds
Source: Front Public Health. 2021 Mar 9;9:622558. doi: 10.3389/fpubh.2021.622558 (PMC7985345; doi:10.3389/fpubh.2021.622558)
Supplement: Supplementary file 1 [file Table_1.pdf]

Open, high-resolution EI+ spectral library of anthropogenic compounds

Elliott J. Price[1,2*], Jiří Palát[2], Kateřina Coufaliková[2], Petr Kukučka[2], Garry Codling[2], Chiara Maria Vitale[2], Štěpán Koudelka[2], Jana Klánová[2]

[1]Faculty of Sports Studies, Masaryk University, Brno, Czech Republic

[2]RECETOX Centre, Masaryk University, Brno, Czech Republic

Supplementary Tables

Supplementary Table 1. Match scores, retention index comparisons, presence on HBM4EU CECscreen and other openly available libraries.

Supplementary Table 1. Match scores, retention index comparisons, presence on HBM4EU CECscreen and other openly available libraries.

Name	NIST Match	HBM4EU CECscreen	Experimental RI	NIST consensus semi non-polar RI	Common with open libraries
1,1-Dibromo-2,3,3,4,4,5-hexachloro-2-cyclopenta-2,4-dien-1-ylcyclooctane			2836.80		
1,2,3,4,6,7,8-Heptachlorodibenzofuran		Y	2904.35	2898	
1,2,3,4,6,7,8-Heptachlorodibenzo-p-dioxin			2956.73	2994	
1,2,3,4,7,8-Hexachlorodibenzofuran		Y	2705.67		
1,2,3,4,7,8-Hexachlorodibenzo-p-dioxin	774	Y	2796.54	2781	
1,2,3,7,8-Pentachlorodibenzofuran	878	Y	2496.11	2507	
1,2,3,7,8-Pentachlorodibenzo-p-dioxin	890	Y	2561.60	2587	
1,2,5,6-Tetrabromocyclooctane	862	Y	2074.80		
1,2,7,9-Tetrachlorodibenzofuran	835	Y	2336.90		
1,2,7,9-Tetrachlorodibenzo-p-dioxin	737		2339.30		
1,2-Benzanthraquinone	929	Y	2588.46	2500	
1,2-Dimethylnaphthalene	882		1432.00	1452	Y
1,3-Dimethylnaphthalene	924	Y	1395.40	1417	
1,4-Chrysenequinone	520	Y	2717.18		
1,4-Dimethylnaphthalene	815	Y	1414.30	1436	
1,4-Naphthoquinone	705	Y	1371.54	1404	
1,5-Dimethylnaphthalene	632	Y	1418.00	1440	
1,8-Dimethylnaphthalene	925	Y	1452.80	1472	
17-alpha-Ethynylestradiol	884	Y	2792.70	2783	
17-beta-Estradiol	769	Y	2723.00	2717	
1-Fluoronaphthalene	891	Y	1196.42	1207	
1-Methylnaphthalene	974		1286.60	1307	Y
1-Methylphenanthrene	932	Y	1967.30	1900	
1-Nitronaphthalene	843	Y	1569.52	1612	Y
2,2',3,3',4,4',5,5',6-Nonachlorobiphenyl	867	Y	2778.40		

Name	NIST Match	HBM4EU CECscreen	Experimental RI	NIST consensus semi non-polar RI	Common with open libraries
2,2',3,3',4,4',5-Heptachlorobiphenyl	928	Y	2566.50	2532	
2,2',3,3',4,5',6,6'-Octachlorobiphenyl	659	Y	2473.00	2518	
2,2',3,4,4',5,5'-Heptachlorobiphenyl	862	Y	2504.78	2484	
2,2',3,4,4',5',6-Heptabromodiphenyl ether	523	Y	3206.14		
2,2',3,4,4',5',6-Heptachlorobiphenyl	927	Y	2386.00	2392	
2,2',3',4,4',5-Hexachloro-3-methoxybiphenyl			2492.51		
2,2',3,4,4',5'-Hexachlorobiphenyl		Y	2356.30	2345	
2,2',3,4',5,5',6-Heptachloro-4-methoxybiphenyl			2592.52		
2,2',3,4,5,5',6-Heptachlorobiphenyl	879	Y	2425.21	2389	
2,2',3,4',5,5',6-Heptachlorobiphenyl	931	Y	2394.20	2378	
2,2',3,4',5,5'-Hexachloro-4-methoxybiphenyl			2554.15		
2,2',3,4,5,5'-Hexachlorobiphenyl	919	Y	2321.77	2315	
2,2',3,4,5'-Pentachlorobiphenyl	864	Y	2162.46	2162	
2,2',3,4',5-Pentachlorobiphenyl	905	Y	2134.21	2074	
2,2',3,5,5',6-Hexachlorobiphenyl	854	Y	2211.16	2198	
2,2',3,5'-Tetrachlorobiphenyl	941	Y	1962.88	1965	
2,2',4,4',5,5'-Hexachlorobiphenyl		Y	2293.50	2300	
2,2',4,5,5'-Pentachlorobiphenyl	820	Y	2135.00	2117	
2,2',5,5'-Tetrachlorobiphenyl	912	Y	1967.40	1933	
2,2',5-Trichlorobiphenyl	892	Y	1753.31	1774	
2,3,3',4',5,5',6-Heptachlorobiphenyl		Y	2504.60	2482	
2,3,3',4,5,6-Hexachlorobiphenyl	897	Y	2357.83	2362	
2,3,3',4',5,6-Hexachlorobiphenyl	866		2360.80	2350	
2,3,3',4',5',6-Hexachlorobiphenyl	900	Y	2356.10	2317	
2,3,3',4,5-Pentachlorobiphenyl	908	Y	2258.70	2207	
2,3,3',4',6-Pentachlorobiphenyl	932	Y	2183.98	2185	

Name	NIST Match	HBM4EU CECscreen	Experimental RI	NIST consensus semi non-polar RI	Common with open libraries
2,3',4,4',5',6-Hexachlorobiphenyl	887	Y	2305.40	2447	
2,3',4,4',5-Pentachlorobiphenyl	926	Y	2262.45	2194	
2,3',4,4'-Tetrachlorobiphenyl	867	Y	2053.00	2056	
2,3,4,5,6-Pentabromobenzyl alcohol			2726.00		
2,3,4,5,6-Pentabromoethylbenzene	913	Y	2323.70		
2,3,4,5,6-Pentabromotoluene	895		2270.30		
2,3,4,5-Tetrabromo-6-chlorotoluene	826	Y	2139.40		
2,3',4,6-Tetrachlorobiphenyl	829	Y	1958.00	1932	
2,3,5,6-Tetrabromo-p-xylene	926	Y	2051.40		
2,3',5',6-Tetrachlorobiphenyl	903	Y	1964.70	1959	
2,3,7,8-Tetrachlorodibenzofuran	835	Y	2314.81	2338	
2,3,7,8-Tetrachlorodibenzo-p-dioxin	868	Y	2347.66	2385	
2,3-Benzofluorene	914	Y	2257.49	2190	
2,3-Dichlorobiphenyl	887	Y	1668.15	1692	
2,4,4'-Trichlorobiphenyl	882	Y	1905.18	1861	
2,4',5-Trichlorobiphenyl	845	Y	1846.20	1859	
2,4,6-Tribromophenol	838	Y	1677.90	1624	Y
2,4,6-Trichlorobiphenyl	888	Y	1796.69		
2,4-D	761		1696.60	1671	
2,4'-Dichlorodiphenyldichloroethane	850	Y	2185.62	2170	
2,4'-Dichlorodiphenyldichloroethylene	895	Y	2107.71	2097	
2,4'-Dichlorodiphenyltrichloroethane	894	Y	2336.20	2261	
2,4-Di-tert-butyl-6-(5-chloro-2H-benzotriazol-2-yl)phenol	752	Y	2652.80		
2,6-Dichloro-4-nitroaniline	696		1755.75	1732	
2,6-Dimethylnaphthalene	855	Y	1381.90	1401	Y
2-Bromoallyl(2,4,6-tribromophenyl) ether			2060.10		

Name	NIST Match	HBM4EU CECscreen	Experimental RI	NIST consensus semi non-polar RI	Common with open libraries
2-Chlorobiphenyl	901	Y	1463.02	1492	
2-Ethylhexyl 2,3,4,5-Tetrabromobenzoate		Y	2834.10		
2-Methylnaphthalene	959	Y	1270.17	1298	Y
2-tert-Butyl-4-methoxyphenol	899	Y	1497.77	1497	
3,3',4,5,5'-Pentachlorobiphenyl	919	Y	2316.20	2266	
4,4'-Dichlorodiphenyldichloroethane	907	Y	2264.41	2257	
4,4'-Dichlorodiphenyldichloroethylene	908	Y	2171.68	2168	
4,4'-Dichlorodiphenyltrichloroethane	911	Y	2348.06	2334	
4-Methoxybenzyl alcohol	908	Y	1285.70	1290	Y
4-Methylbenzophenone	768	Y	1786.55		Y
4-tert-Octylphenol	829	Y	1601.60	1601	
7,12-Dimethylbenz[a]anthracene	855	Y	2819.70	2712	
Acenaphthene	898	Y	1528.33	1486	Y
Acenaphthylene	781	Y	1500.98	1454	Y
Acephate	143	Y	1464.27	1420	Y
Acibenzolar-S-methyl	861	Y	1900.60		
Alachlor	689	Y	1889.49	1894	
Aldrin	903	Y	1940.09	1955	
Allyl 2,4,6-tribromophenyl ether	773	Y	1753.50		
alpha-1,2,3,4,5,6-Hexachlorocyclohexane	822	Y	1732.42	1693	
alpha-1,2-Dibromo-4-(1,2-dibromoethyl)cyclohexane	865	Y	1992.50		
alpha-Amylcinnamaldehyde	951	Y	1651.24	1659	Y
alpha-Amylcinnamyl alcohol	898		1688.80	1675	
alpha-Hexylcinnamaldehyde	951		1751.05	1750	Y
Ametryn	867	Y	1892.00	1898	
Aminocarb	925	Y	1743.20	1770	

Name	NIST Match	HBM4EU CECscreen	Experimental RI	NIST consensus semi non-polar RI	Common with open libraries
Amitraz	946		2563.60	2559	
Anthanthrene	590	Y	3283.45	3185	
Anthracene	903	Y	1844.37	1775	Y
anti-Dechlorane plus		Y	3412.90		
Atrazine	604	Y	1730.38	1746	
Azoxystrobin		Y	3076.10	3076	
Benalaxyl	831	Y	2388.60	2313	
Bendiocarb	886	Y	1647.10	1667	
Benzanthracene/Benzophenanthrene		Y	2469.98	2441	
Benzo(a)pyrene	945	Y	2858.04	2790	Y
Benzo(g,h,i)perylene	954	Y	3243.52	3150	
Benzo(k)fluoranthene	943	Y	2777.20	2748	Y
Benzo[b]naphtho[2,1-d]thiophene	882	Y	2419.34	2359	
Benzo[e]pyrene	953		2843.99	2771	
Benzo[ghi]fluoranthene	902	Y	2407.20		
Benzofluoranthene		Y	2769.96		
Benzophenone	846	Y	1659.58	1666	Y
Benzoximate		Y	1925.60		
Benzyl alcohol	868	Y	1033.20	1036	Y
Benzyl benzoate	895	Y	1776.60	1762	Y
Benzyl cinnamate	882	Y	2110.20	2135	
Benzyl salicylate	929	Y	1882.40	1869	Y
beta-1,2-Dibromo-4-(1,2-dibromoethyl)cyclohexane	843	Y (alpha above)	2006.10		
beta-Hexachlorocyclohexane	911	Y	1775.60	1741	
Bifenazate	869	Y	2450.20	2515	
Bifenthrin	870		2464.20	2470	

Name	NIST Match	HBM4EU CECscreen	Experimental RI	NIST consensus semi non-polar RI	Common with open libraries
Bisphenol A	805		2195.20	2108	
Bitertanol_isomer1	860	Y	2714.60	2637	
Bitertanol_isomer2	841	(as above)	2725.00	2648	
Boscalid	774	Y	2830.20		
Bromuconazole_isomer1	848	Y	2485.50	2487	
Bromuconazole_isomer2	796	(as above)	2527.80	2487	
Bumetrizole		Y	2541.48		
Bupirimate	816	Y	2206.20	2202	
Buprofezin	902	Y	2197.80	2195	
Butafenacil	848	Y	2741.70		
Butylated hydroxytoluene	754	Y	1516.07	1513	
Camphor	890	Y	1156.46	1142	Y
Carbaryl	892	Y	1908.00	1901	Y
Carbetamide	853	Y	2009.30	1974	
Carbofuran	939	Y	1703.80	1735	
Carbofuran phenol	941	Y	1292.20	1286	
Carboxin	854	Y	2221.00	2189	
Carfentrazone-ethyl		Y	2335.60	2327	
Cashmeran	905	Y	1493.70	1508	
Celestolide	907	Y	1696.07	1707	
Chlorferone		Y	2275.41		
Chlorpyrifos	750	Y	1977.26	1973	
Chlorpyrifos oxon	822	Y	1968.14	1973	
Cinnamaldehyde	971		1278.08	1274	Y
Cinnamyl alcohol	920		1309.50	1313	Y
cis-Allethrin	846		2071.80		

Name	NIST Match	HBM4EU CECscreen	Experimental RI	NIST consensus semi non-polar RI	Common with open libraries
cis-Chlordane	895	Y	2112.09	2111	
cis-Cyfluthrin_isomer1	650	Y	2764.18		
cis-Cyfluthrin_isomer2		(as above)	2787.22		
cis-Cypermethrin_isomer1		Y	2809.30	2574	
cis-Cypermethrin_isomer2	730	(as above)	2831.80	2774	
cis-Cyphenothrin		Y	2652.77		
cis-Fenvalerate	819	Y	2945.03	2847	
cis-Heptachlor epoxide	872		2026.06	2059	
cis-Permethrin	825		2686.26	2648	
cis-Phenothrin	863	Y	2517.10	2524	
cis-Prallethrin	823	Y	2096.98	2136	
cis-Resmethrin	810		2394.50	2394	
cis-Tetramethrin	716		2448.80		
Coronene	688	Y	3652.60	3498	Y
Coumarin	957	Y	1446.80	1441	Y
Cyazofamid	528	Y	2429.80	2388	
Cycluron	856	Y	1752.20	1760	
Cyproconazole_isomer1	868	Y	2235.30	2236	
Cyproconazole_isomer2	860	(as above)	2239.80	2339	
delta-Hexachlorocyclohexane	829	Y	1842.60	1801	
delta-Iraldeine	802		1500.30	1506	
Deltamethrin	620	Y	3045.50	3059	
Diazinone	650	Y	1791.02	1791	
Dibenzanthracene		Y	3190.46		
Diclobutrazol	818		2205.20	2191	
Dicofol		Y	2379.70	2467	

Name	NIST Match	HBM4EU CECscreen	Experimental RI	NIST consensus semi non-polar RI	Common with open libraries
Dicrotophos	777	Y	1656.09	1656	
Diethofencarb	794	Y	1966.60	1964	
Difenoconazole_isomer1	757	Y	3010.30	3017	
Difenoconazole_isomer2	756	(as above)	3016.20	3025	
Diflubenzuron		Y	1298.90		
Dimethachlor	876	Y	1854.55	1863	
Dimethoate	819	Y	1702.17	1720	Y
Dimethomorph _isomer1		Y	3076.97	3080	
Dimethomorph _isomer2		(as above)	3111.30	3159	
Diniconazole	734		2263.70	2370	
Dioxacarb	870	Y	1872.81	1850	
Drometrizole	850		2085.60		
Endosulfan	785	Y	2024.49	2114	
Endosulfan sulphate	825	Y	2328.83	2328	
Endrin	905		2205.33	2211	
Endrin aldehyde	855		2275.78	2261	
Endrin ketone	914		2430.05	2426	
Enilconazole	747		2158.30	2161	
Enzacamene			2079.00		
Epoxiconazole	855	Y	2463.90	2425	
epsilon-Hexachlorocyclohexane		Y	1865.93		
Estragole	934	Y	1202.15	1196	Y
Estrone	929	Y	2701.20	2694	Y
Ethiofencarb	842	Y	1850.20	1837	
Ethiprole		Y	2434.50		
Ethofumesate	866	Y	1954.40	1938	

Name	NIST Match	HBM4EU CECscreen	Experimental RI	NIST consensus semi non-polar RI	Common with open libraries
Etoxazole	881	Y	2491.50	2489	
Eucalyptol		Y	1037.80	1032	Y
Eugenol	957	Y	1355.58	1357	Y
Fenamidone	812	Y	2516.10	2499	
Fenarimol	820	Y	2638.90	2579	
Fenazaquin	918	Y	2559.00	2506	
Fenhexamid	887	Y	2416.60		
Fenobucarb	945	Y	1582.00	1608	
Fenoxaprop-ethyl	779	Y	2684.27		
Fenoxycarb	789	Y	2487.80	2459	
Fenpropathrin	824	Y	2481.19	2495	
Fenpropimorph_isomer1	823	Y	1911.80		
Fenpropimorph_isomer2	833	(as above)	1954.80		
Fipronil	774	Y	2045.70	2052	
Flucythrinate_isomer1	834		2833.70	2844	
Flucythrinate_isomer2	731		2862.27	2844	
Fludioxonil	863	Y	2240.80	2169	
Flufenacet	893	Y	1983.70		
Fluoranthene	970	Y	2102.69	2054	Y
Fluquinconazole	905	Y	2744.90	2729	
Flusilazole	750	Y	2204.80	2192	
Flutolanil	933	Y	2130.00	2147	
Flutriafol	919	Y	2126.70	2155	
Fuberidazole	906	Y	1921.20	1891	
Furalaxyl	844	Y	2065.00	2074	
Furathiocarb	778	Y	2521.00	2508	

Name	NIST Match	HBM4EU CECscreen	Experimental RI	NIST consensus semi non-polar RI	Common with open libraries
Galaxolide	891	Y	1830.61	1851	
Geranial	872	Y	1268.00	1270	
Heptachlor	909	Y	1871.08	1887	
Hexabromobenzene	703	Y	2475.30	2473	
Hexachlorobenzene	930	Y	1744.46	1707	Y
Hexaconazole	843	Y	2125.50	2150	
Hydroxychrysene	890	Y	2864.21		
Hydroxyfluorenone	901	Y	1751.11		
Indeno[1,2,3-cd]pyrene	826	Y	3177.05	3095	
Indoxacarb	756	Y	3019.30		
Ipconazole	874	Y	2595.00		
Iprovalicarb isomer 1	842	Y	2168.30		
Iprovalicarb isomer 2	837	(as above)	2205.10		
Isocarbophos	683	Y	2005.30		
Isodecyl diphenyl phosphate	835		2427.00		
Isoeugenol	849	Y	1452.11	1450	Y
Isomethyl-alpha-ionone	929		1479.90	1480	
Isoprocarb	898	Y	1511.80	1532	
Kresoxim-methyl	931	Y	2211.30	2202	
lambda-Cyhalothrin	764	Y	2585.27	2565	
Lilial	927	Y	1532.00	1534	
Limonene	879	Y	1032.90	1030	Y
Lindane	889	Y	1798.00	1754	Y
Linuron	689	Y	1954.10	1946	
Malathion	877	Y	1964.44	1959	Y
Mefenacet	885	Y	2584.70	2566	

Name	NIST Match	HBM4EU CECscreen	Experimental RI	NIST consensus semi non-polar RI	Common with open libraries
Mepanipyrim	837	Y	2096.60	2134	
Mepronil	716	Y	2296.40	2298	Y
Metalaxyl	842	Y	1906.80	1903	
Metazachlor	685	Y	2036.81	2043	
Metconazole	869	Y	2520.10		
Methabenzthiazuron		Y	1648.30		
Methiocarb	720	Y	1946.60	1932	
Methomyl		Y	1568.50	1532	
Methoprotryne	750	Y	2207.70	2193	
Methoxychlor	904	Y	2475.70	2453	
Methyl parathion	818	Y	1890.00	1876	Y
Methyleugenol	943	Y	1398.78	1402	
Metobromuron	869	Y	1843.90	1837	
Metolachlor	873	Y	1956.20		
Metribuzin	769	Y	1876.10	1864	
Mevinphos	774	Y	1445.50	1419	
Mexacarbate	929	Y	1812.30		
Mirex	933	Y	2586.68	2530	
Monocrotophos	870	Y	1723.10	1662	
Monolinuron	920	Y	1705.30	1740	
Myclobutanil	923	Y	2197.70	2198	Y
Nuarimol	843	Y	2440.10	2374	
Octachlorodibenzofuran		Y	3099.39	3147	
Octachlorodibenzo-p-dioxin		Y	3097.63	3197	
Octrizole	757	Y	2571.94		
Oxadixyl	743	Y	2295.70	2268	

Name	NIST Match	HBM4EU CECscreen	Experimental RI	NIST consensus semi non-polar RI	Common with open libraries
Paclobutrazol	852		2084.00	2109	
para-Terphenyl	897		2207.51	2152	
Penconazole	844	Y	2037.70	2045	
Pendimethalin	804	Y	2044.58	2038	
Pentabromobenzene		Y	2083.60		
Pentachlorobenzene	864	Y	1554.64	1511	
Perylene	935	Y	2886.93	2815	Y
Perylene_2H12	818		2875.96		
Phantolide	869	Y	1737.42		
Phenanthrene	916	Y	1832.86	1775	Y
Phenanthrene_2H10	866	Y	1827.10	1784	
Phosmet	915		2460.21	2445	
Picoxystrobin	937	Y	2105.40		
Piperonyl butoxide	874	Y	2431.90	2396	
Pirimicarb	778	Y	1829.60	1837	
Praziquantel	916	Y	2970.57		
Progesterone	906	Y	2892.70		Y
Promecarb	916	Y	1652.70	1687	
Prometon	867	Y	1691.20	1727	
Prometryn	875	Y	1894.10	1905	
Propargite_isomer1	821	Y	2426.80	2387	
Propargite_isomer2	860	(as above)	2429.50	2387	
Propham	912	Y	1428.80	1453	
Propiconazole_isomer1	726	Y	2404.40	2336	
Propiconazole_isomer2	831	(as above)	2412.00	2351	
Propoxur	909	Y	1588.60	1606	

Name	NIST Match	HBM4EU CECscreen	Experimental RI	NIST consensus semi non-polar RI	Common with open libraries
Pyracarbolid	858	Y	2019.90		
Pyraclostrobin	819	Y	2964.19	2967	
Pyrene	969	Y	2154.49	2091	Y
Pyridaben	892	Y	2723.80	2648	
Pyrimethanil	765	Y	1769.70	1793	
Pyriproxyfen	848	Y	2557.70	2574	
Quinoxyfen	762	Y	2403.20	2347	
Retene	852	Y	2236.17	2198	
Rotenone	644	Y	3213.70	3242	
Safrole	910	Y	1296.00	1287	Y
Secbumeton	847	Y	1805.00	1804	
Siduron	666	Y	2153.50	2132	
Simetryn		Y	1890.90	1902	
Spirodiclofen		Y	2708.90	2690	
Spiromesifen	732	Y	2456.30		
Spirotetramat			2767.80		
Spiroxamine_isomer1	843	Y	1859.10	1896	
Spiroxamine_isomer2	811	(as above)	1907.20	1949	
Sulfentrazone	867	Y	2507.80		
syn-Dechlorane plus		Y (anti above)	3373.90		
Tebuconazole	830	Y	2434.30	2391	
Tebufenpyrad	899	Y	2493.50	2501	
Tebuthiuron	857	Y	1523.20	1504	
Tefluthrin	812	Y	1811.79		
Temephos	600	Y	3218.50	3097	
Terbufos	896	Y	1748.90	1768	

Name	NIST Match	HBM4EU CECscreen	Experimental RI	NIST consensus semi non-polar RI	Common with open libraries
Terbumeton	876	Y	1721.00	1738	
Terbuthylazine	839	Y	1758.80	1771	
Terbutryn		Y	1927.70	1928	
Testosterone	926	Y	2733.00		Y
Tetraconazole	782	Y	2000.40	1998	
Thiabendazole	869	Y	2074.20	2052	Y
Thiobencarb	882	Y	1957.00	1974	
Thiofanox		Y	1214.20	1277	
Tonalide	807	Y	1845.27	1843	
trans-Allethrin	896		2075.10	2062	
trans-Chlordane	856		2079.00	2078	
trans-Cyfluthrin_isomer1	763	Y	2779.20		
trans-Cyfluthrin_Isomer2	726	(as above)	2793.60		
trans-Cypermethrin_isomer1	797	Y	2817.29	2766	
trans-Cypermethrin_isomer2	699	(as above)	2841.71	2777	
trans-Cyphenothrin		Y (cis above)	2656.66		
trans-Fenvalerate	707	Y (cis above)	2965.30	2847	
Transfluthrin	881	Y	1902.49	1910	
trans-Permethrin	841	Y	2701.88	2657	
trans-Phenothrin	831	Y	2532.40	2524	
trans-Prallethrin	844	Y (cis above)	2102.15	2136	
trans-Resmethrin	831		2407.62	2394	
trans-Tetramethrin	895	Y	2468.00		
Traseolide	788	Y	1830.96	1830	
Triadimefon	771	Y	1987.90	1986	
Triadimenol_isomer1	885	Y	2056.90	2076	

Name	NIST Match	HBM4EU CECscreen	Experimental RI	NIST consensus semi non-polar RI	Common with open libraries
Triadimenol_isomer2	708	(as above)	2070.70	2083	
Triclosan	784	Y	2122.80	2114	
Tricyclazole	762	Y	2214.60	2158	
Trifloxystrobin	863		2399.00	2339	
Triflumizole	755		2061.50	2080	
Trifluralin	811	Y	1666.18	1669	Y
Tri-m-cresyl phosphate	840	Y	2686.60	2664	
Tri-n-butyl-phosphate	890	Y	1628.13	1655	
Tri-o-cresyl phosphate	945	Y	2611.20	2555	
Tri-p-cresyl-phosphate	892		2784.00		
Triphenyl phosphate	833	Y	2400.00	2387	Y
Triphenylene	917	Y	2463.80	2416	Y
Tris(1,3-dichloro-2-propyl)phosphate	825	Y	2338.54	2251	
Tris(1-chloro-2-propyl) phosphate	679	Y	1783.15	1816	
Tris(2-butoxyethyl) phosphate	842	Y	2407.00	2421	
Tris(2-chloroethyl) phosphate	912	Y	1756.00	1777	
Tris(2-ethylhexyl) phosphate	836	Y	2465.00	2463	
Tris(3,5-xylenyl)phosphate			2906.40		
Tris(4-tert-butylphenyl) phosphate	800	Y	3270.93		
Tris(isopropylphenyl)phosphate	868		2815.13		
Triticonazole	828		2553.00		
Vamidothion	724	Y	2125.30	2109	
Zoxamide	633	Y	2453.90	2428	
