# Supplementary material for: Open, High-Resolution EI+ Spectral Library of Anthropogenic Compounds
Source: Front Public Health. 2021 Mar 9;9:622558. doi: 10.3389/fpubh.2021.622558 (PMC7985345; doi:10.3389/fpubh.2021.622558)
Supplement: Supplementary file 2 [file Data_Sheet_1.pdf]

Open, high-resolution EI+ spectral library of anthropogenic compounds

Elliott J. Price[1,2*], Jiří Palát[2], Kateřina Coufaliková[2], Petr Kukučka[2], Garry Codling[2], Chiara Maria Vitale[2], Štěpán Koudelka[2], Jana Klánová[2]

[1]Faculty of Sports Studies, Masaryk University, Brno, Czech Republic

[2]RECETOX Centre, Masaryk University, Brno, Czech Republic

Supplementary Figures

Supplementary Figure 1. Distribution curves showing physicochemical range of library**.**

Supplementary Figure 2. Example matches of library spectrum.

Supplementary Figure 3. Example analysis using compound database.

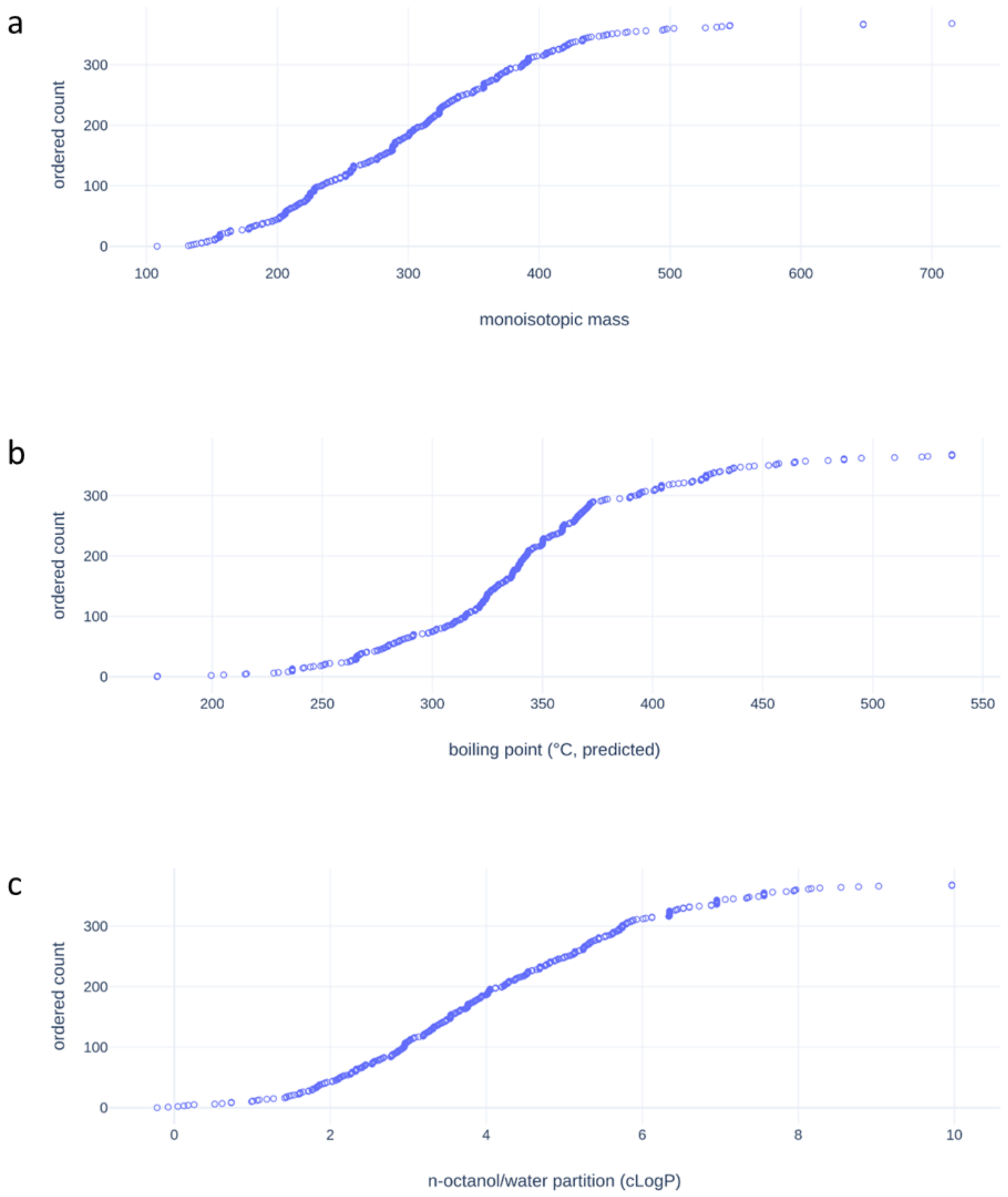

Supplementary Figure 1. Distribution curves showing physicochemical range of library. Properties (a) monoisotopic mass, (b) predicted boiling point and (c) predicted LogP for each compound (○) are plotted.

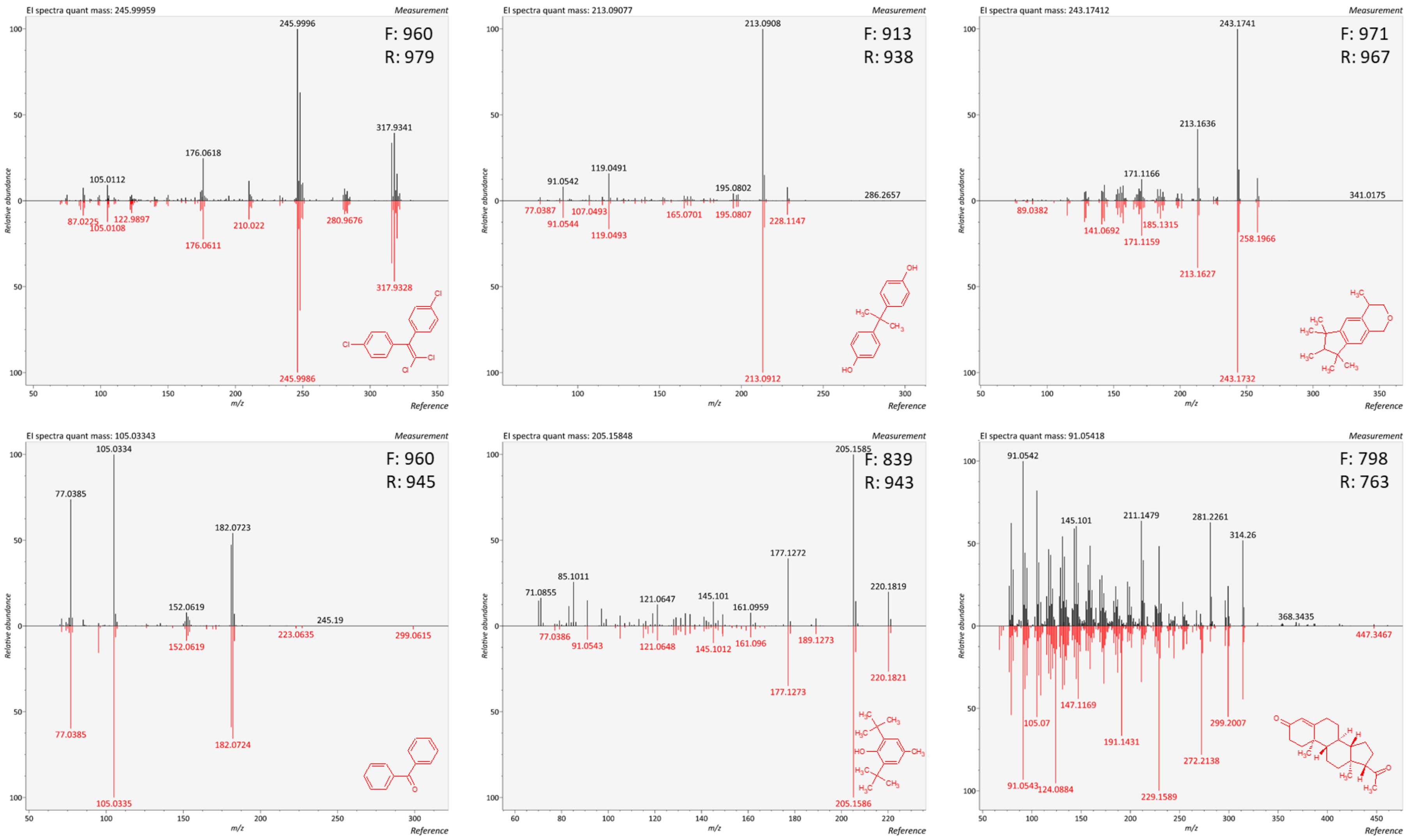

Supplementary Figure 2. Example matches of library spectrum (red) to deconvoluted spectra (black) in non-fortified house dust or human serum matrix (top and bottom, respectively). Match undertaken within MS-DIAL software, showing forward (F) and reverse (R) scores. Compounds clockwise (from top left), 4,4'-dichlorodiphenyldichloroethylene (p,p'-DDE); bisphenol A (BPA); galaxolide (HHCB); benzophenone; butylated hydroxytoluene (BHT) and progesterone, drawn in ACD/ChemSketch.

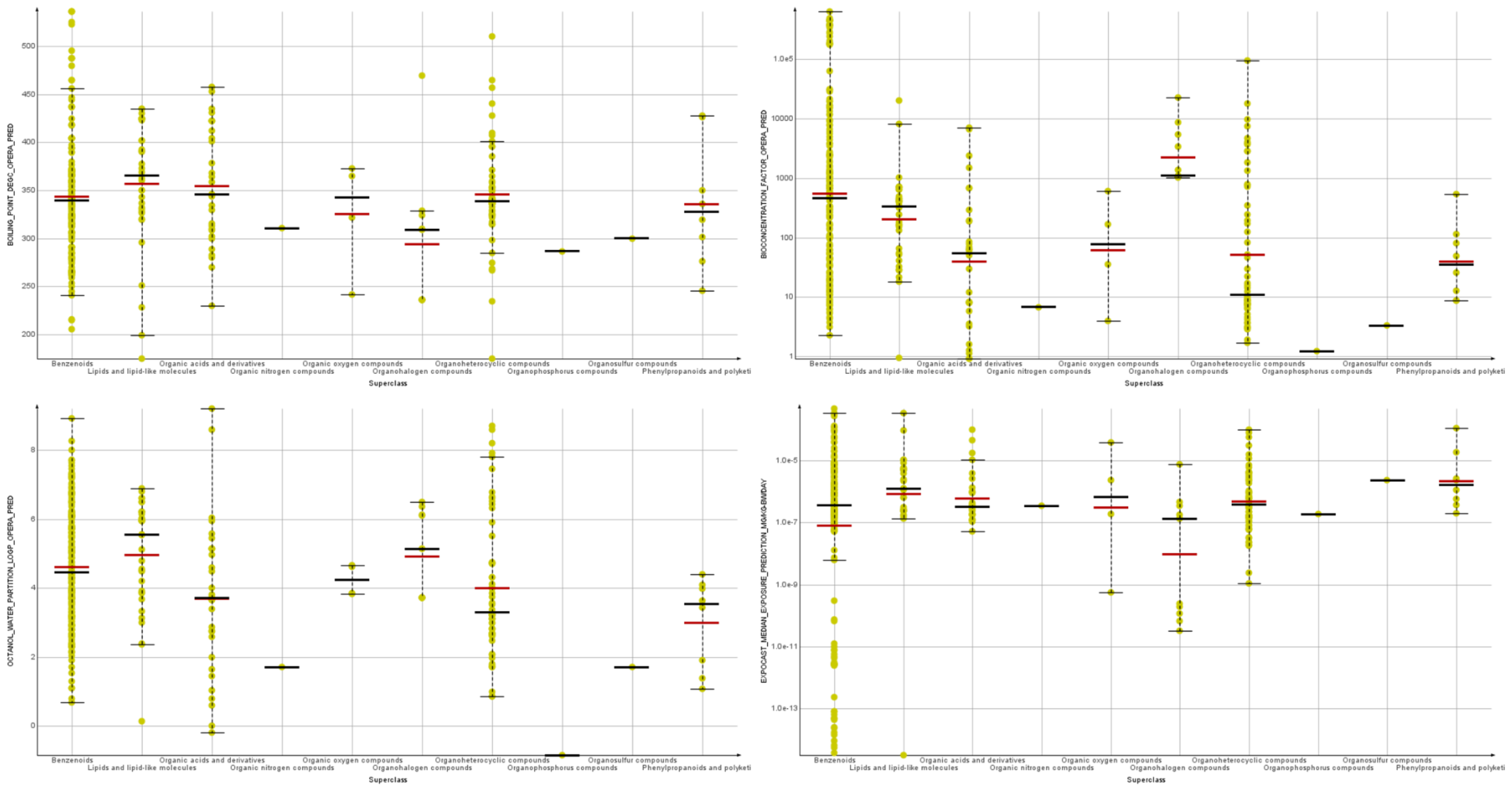

Supplementary Figure 3. Example analysis using compound database to compare physicochemical properties per structural chemical classification. Whisker plots were generated within DataWarrior following direct import of the SDF compound database, excluding structures lacking predicted physico-toxic properties.
